# Nerve growth factor-chondroitin sulfate/hydroxyapatite-coating composite implant induces early osseointegration and nerve regeneration of peri-implant tissues in Beagle dogs

**DOI:** 10.1186/s13018-020-02177-5

**Published:** 2021-01-13

**Authors:** Jun Ye, Bo Huang, Ping Gong

**Affiliations:** 1 Department of Prosthodontics, School and Hospital of Stomatology, Tongji University and Shanghai Engineering Research Center of Tooth Restoration and Regeneration, Shanghai, 200072 People’s Republic of China; 2 State Key Laboratory of Oral Diseases, General Dentistry, West China Hospital of Stomatology, Sichuan University, Chengdu, 610041 People’s Republic of China; 3 State Key Laboratory of Oral Diseases, Department of Oral Implant, West China School of Stomatology, Sichuan University, Chengdu, 610041 People’s Republic of China

**Keywords:** NGF-CS/HA-coating composite titanium, Bone marrow mesenchymal stem cells, Osteogenesis differentiation, Neuronal differentiation

## Abstract

**Background:**

Osseointegration is the premise of the chewing function of dental implant. Nerve growth factor (NGF), as a neurotrophic factor, can induce bone healing. However, the influence of NGF-chondroitin sulfate (CS)/hydroxyapatite (HA)-coating composite implant on the osseointegration and innervations is still not entirely clear.

**Materials and methods:**

NGF-CS/HA-coating composite implants were prepared using the modified biomimetic method. The characteristics of NGF-CS/HA-coating implants were determined using a scanning electron microscope. After NGF-CS/HA-coating implants were placed in the mandible of Beagle dogs, the early osseointegration and innervation in peri-implant tissues were assessed through X-ray, Micro-CT, maximal pull-out force, double fluorescence staining, toluidine blue staining, DiI neural tracer, immunohistochemistry, and RT-qPCR assays.

**Results:**

NGF-CS/HA-coating composite implants were made successfully, which presented porous mesh structures with the main components (Ti and HA). Besides, we revealed that implantation of NGF-CS/HA-coating implants significantly changed the morphology of bone tissues and elevated maximum output, MAR, BIC, and nerve fiber in the mandible of Beagle dogs. Moreover, we proved that the implantation of NGF-CS/HA-coating implants also markedly upregulated the levels of NGF, osteogenesis differentiation, and neurogenic differentiation-related genes in the mandible of Beagle dogs.

**Conclusion:**

Implantation of NGF-CS/HA-coating composite implants has significant induction effects on the early osseointegration and nerve regeneration of peri-implant tissues in the mandible of Beagle dogs.

**Supplementary Information:**

The online version contains supplementary material available at 10.1186/s13018-020-02177-5.

## Introduction

With the development of oral implantology, implant prostheses have become vital methods for the effective treatment of dentition defects and loss and restoration of oral function [1]. The ideal dental materials should have a variety of properties, such as good biological properties, good mechanical properties, economy, and easy transport and storage [2, 3]. Since implant placement typically requires a 3–6-month osseointegration cycle, the combination of implant and bone interface is the premise of successful dental implant restoration [4]. It has become the key to shorten the osseointegration period and improve the osseointegration rate of the implants by modifying the surface of the implants in oral implants. The surface characteristics of dental implants can affect the biological reaction after implant implantation and directly affect the bone healing rate, bone binding rate, and bone binding strength of the interface, which is very important for the normal exercise of implant function [5]. Research proved that the surface characteristics of dental implants can directly affect the speed of bone healing, the rate of bone bonding, and the strength of bone bonding [6]. The surface modification of implants is to change the surface morphology and composition of implants by means of physical, chemical, and biological methods to promote bone-tissue growth around the implants [7]. A study demonstrated that the calcium phosphate coating was similar to bone tissue in composition and had good biocompatibility and bone conductivity [8]. Therefore, the addition of bone-induced bioactive molecules to the surface of titanium implants may contribute significantly to the biological functionalization of titanium surface.

Bone grafting is one of the most commonly used options for bone defect treatment, and new strategies such as gene therapy, polytherapy by using scaffolds, healing promotive factors and stem cells, and three-dimensional printing have been developed as potential stages for treating bone defects [9, 10]. Studies verified that the dysdifferentiation of osteoblasts around the implants and the damage of new bone formation can significantly affect the normal healing of the implant-bone interface, resulting in a longer healing and repair time after implantation [11, 12]. Besides, researches demonstrated that there are significant differences in biomechanics and neurophysiology between implants and natural teeth [13–15]. In recent years, more and more scholars have applied the exogenous substances to promote bone binding of implants, such as BMP-2 [16, 17], phosphorylated chitin [18], HIF-1alpha [19], and insulin-like growth factor binding protein-3 [20]. Unfortunately, most investigations of dental implants, including the previously mentioned surface modification technologies, focus on growth factors and their related signaling pathways, while few studies consider the regulation of nerves and neurohumor. Sympathetic nerves are widely distributed in bone tissue and play an important role in the regulation of bone formation via a number of adrenergic receptors in osteoblasts [21]. For example, hypothalamic leptin can react with the sympathetic nervous system, thereby regulating bone formation [22, 23]. Many researchers have successfully regulated the bone formation process by using drugs or by transecting the sympathetic nerve of the bone, though the mechanisms of action for these methods of regulation are not clearly understood [24, 25]. These studies showed that sympathetic nervous regulation has great potential to promote the formation of implant-bone osseointegration.

The nerve growth factor (NGF) has the dual biological functions of nourishing neurons and promoting neurite growth [26]. NGF is critical in the regulations of functional characteristics, such as development, differentiation, growth, and regeneration in central and peripheral neurons, which can effectively promote osseointegration around implants [27, 28]. The NGF can enhance the activity of osteocytes and promote the differentiation and mineralization of osteoblasts, peripheral nerves, and vascularization in the process of implant-bone binding [29–31]. Research has testified that local injection of NGF could increase bone formation during mandibular distraction osteogenesis, indicating that NGF plays an essential role in bone regeneration [32]. Our previous study showed that NGF-CS/HA-coating composite titanium has significant promoting effects on the differentiation of BMSCs into osteoblast and neural cells in vitro [33]. Therefore, NGF can effectively promote bone healing around implants and thus shorten the time of osseointegration in oral implants.

Currently, the application methods of NGF in bone and nerve repair around implants mainly include direct injection, sustained release carrier, and improvement of implant surface coating structure [34]. Direct injection can easily result in loss of NGF activity [35]. Collagen, polymer polymers, collagen/nanometer hydroxyapatite, and miniature osmotic pumps have been widely used as the sustained-release carriers [29, 36, 37], while the ideal carrier material requires a variety of properties, such as biocompatibility, strength, affinity with NGF, biodegradability, toxicity, etc. The modification of the coating structure on the implant surface has become a novel method to improve the slow-release effect of NGF [38]. It was reported that the HA titanium implant was obtained by immersing the titanium implant in simulated body fluids (SBF); NGF was mixed with chondroitin sulfate (CS) and freeze-dried to form NGF-CS nanoparticles; NGF-CS/HA titanium implant was then formed by soaking the NGF-CS nanoparticles and the titanium implant in calcium phosphate solution [39]. NGF-CS/HA titanium implant has also been proven to slowly release active NGF [40]. However, the NGF-CS/HA titanium implant is still in the experimental stage. The clinical application of NGF in oral implant is still lacking. Therefore, it is the future trend that NGF will be widely applied in oral cavity.

In our study, we implanted NGF-CS/HA composite coating implant into the mandible of Beagle dogs to observe the effects of NGF-CS/HA composite coating on early bone binding and nerve regeneration around the implant in vivo.

## Materials and methods

### Titanium sheet

The titanium sheets (Ti6Al4V) with 14 mm in diameter and 1 mm in thickness were acquired from the National Engineering Research Center for Biomaterials, Sichuan University. All the surfaces of the titanium sheets used in the experiment were treated with sandblast-acid erosion (SLA) and alkali-heat.

### Preparation of NGF-CS complex

NGF (Sigma-Aldrich, St. Louis, MO, USA) and CS (Solarbio, cat. no. C9160) were dissolved in a buffer solution (pH = 7.4) in a 1:1 ratio and stirred at 4 °C for 30 min under aseptic conditions. Then the NGF-CS complex was freeze-dried for 24 h.

### Preparations of HA and NGF-CS/HA composite coatings

For the HA coating, the implant was placed into 5 × SBF (simulated body fluid) and bathed at 37 °C for 24 h under aseptic conditions, and the procedure was repeated twice. For the NGF-CS/HA composite coating, the implant was placed into 5 × SBF at 37 °C for 24 h and then 5 ml 5 × SBF containing 5 mg/l NGF-CS complex at 37 °C for 24 h under aseptic conditions.

### Scanning electron microscope (SEM)

In line with the previous researches [41, 42], the surface morphology of HA and NGF-CS/HA composite coatings were observed through SEM.

### Detection of adhesion force

The acoustic emission scratch tester (Revetest, CSM Instruments, Switzerland) was adopted to analyze the critical in the HA (*n* = 3) and NGF-CS/HA composite coatings (*n* = 3).

### Component analysis

The Philips analytical PC-APD X-ray diffractometer (Philips Co., Netherlands, PW 1840) was utilized to determine the main components of the HA (*n* = 3) and NGF-CS/HA composite coatings (*n* = 3). And the range of CuKα diffraction (40 kV and 30 mA) was 10–80°.

### Animals

A total of 6 healthy adult male Beagle dogs (15 months old and weigh 13–15 kg) in this study were provided by the experimental animal center of Sichuan University. And animal feeding, surgery, and specimen cutting were all carried out in this experimental animal center. All animal experiments have been approved by the ethics committee of Sichuan University (WCHSIRB-D-2014-109).

### Establishment of experimental animal models

The Beagle dogs were anesthetized by intravenous injection of 3% sodium pentobarbital (30 mg/kg). The Beagle dogs were placed on the operating table in the lateral position. After disinfection with 2.5% povidone-iodine and 75% alcohol, the four mandibular premolars on both sides were extracted minimally, and the gums were tightly sutured. After 6 months, 4 NGF-CS/HA-coating composite implants were implanted in the right mandibular premolar region, and 4 HA-coating implants were implanted in the left mandibular premolar. The observation time points were 2 weeks, 4 weeks, and 8 weeks as previous studies indicated [43, 44], and there were 2 animals at each time point. The Beagle dogs were given intramuscular antibiotics to prevent infection 3 days after the operation.

### Measurement of mineral apposition rate (MAR)

All Beagle dogs were subcutaneously injected with alizarin red at 40 mg/kg on the 13th and 14th days before execution; then the Beagle dogs were subcutaneously injected with calcitrine at 10 mg/kg on the 3rd and 4th days prior to execution. The fluorescence results were examined under a fluorescence microscope. The Image-Pro Plus analysis system was applied to measure the distance between the two labeled fluorescence lines: MAR = distance between the two labeled fluorescence lines (*D*)/time between the two injections (*t*).

### DiI neural tracer

Ten days before execution, the Beagle dogs were anesthetized and the medial sides of the bilateral mandibular angle were disinfected. The inferior alveolar nerve of the mandibular nerve in Beagle dogs was injected with the 4 μl carboxyblue fluorescent agent (DiI, 4 mg/ml) for 15 min, and the incision was closed. After being sacrificed, hard tissue sections with implants were prepared, and the results were observed under a laser confocal fluorescence microscopy.

### Quantitative real-time PCR (RT-qPCR) assay

The implants with thin layers of bone tissue were removed using a ring bone drill. The obtained thin bone tissues on the surface of the implants were crushed and added with Trizol reagent (Invitrogen, Shanghai, China) to extract the total RNAs. Then cDNAs were synthesized by the Reverse Transcription kit (Takara, Japan) using 1 μg RNAs from each sample. Gene expression was detected through BestarTM qPCR Master Mix (DBI Bioscience, China, cat. no. #2043). RT-PCR system was 10 μl Bestar SybrGreen qPCR master mix, 0.5 μl forward primer, 0.5 μl reverse primer, 1 μl cDNAs, and 8.0 μl ddH_2_O. The thermo cycling conditions were as follows: 95 °C for 2 min, followed by 40 cycles of 95 °C for 10 s, 60 °C for 34 s, and 72 °C for 30 s. The gene expression was quantified by the 2^−△△^Cq method [45]. The sequences of primers are displayed in Table 1.

**Table 1:** The sequences of primers in the qRT-PCR assay

ID	Sequence (5′-3′)
GAPDH	Forward: AAGGTCGGAGTCAACGGATTT
GAPDH	Reverse: GGCATCAGCAGAAGGAGCAG
TrkA	Forward: CTCTACCGCAAGTTCACCACG
TrkA	Reverse: TGATGCACTCAATCGCCTCG
p75	Forward: CAACCTCATCCCTGTCTACTGCT
p75	Reverse: GGCTCCTTGCTTGTTCTGCTT
OCN	Forward: GTGCTGAATCCCGCAAAGG
OCN	Reverse: CATACTTCCCTCTTGGGCTCC
Runx-2	Forward: GACCAGCAGCACTCCATATCTCT
Runx-2	Reverse: CTTCCATCAGCGTCAACACCA
Nestin	Forward: CTTGCTGTTGGCACCCTTCC
Nestin	Reverse: CCAGGACACTCACGCACGAA
NF	Forward: AAGAAGCCAAACCCAAAGAGAAG
NF	Reverse: GGGTCTTCTCCTCCTTGACATCTT
Tubulin β-4	Forward: TTCATCGGCAACAGCACAGC
Tubulin β-4	Reverse: GGTACTCAGACACCAGGTCATTCA

### X-ray examination

The specimens at each time point were photographed and observed by an X-ray film using the method of parallel projection. The operating voltage was 65 kV, the current was 7 mA, and the exposure time was 0.1 s.

### Micro-computed tomography (Micro-CT)

Micro-CT scanning analysis system (Y. Cheetah, YXLON International GmbH, Germany) was applied with the parameters voltage 90 kV, current 39 μA, projection number 450, and integral time 0.6 s. The implant and surrounding bone tissue were reconstructed in three dimensions after scanning. The range of interest (ROI) was selected with the implant as the axis. Parameters included bone volume fraction (BVF; %), trabecular thickness (Tb.Th; μm), trabecular number (Tb.N; mm^−1^), and trabecular spacing (Tb.Sp; μm).

### Detection of maximal pull-out force

A universal material testing machine (Model 3365, Instron, Norwood, MA) was applied to confirm the maximal pull-out force at the speed of 1 mm/min.

### Preparation of hard tissue sections

Bone tissues and the coated titanium implants were cut and fixed at 4% paraformaldehyde for 48 h. The samples were dehydrated using 60%, 80%, 90%, and 100% ethanol; embedded for 24 h; and sliced into100 μm thickness using E300CP diamond. Finally, the hard tissue sections were ground with the EXAKT 400 CS microchip grinder (about 60 μm thickness): BIC% = The total length of the interface between the implant and bone matrix/The total length of the contour of the implant embedded × 100%.

### Toluidine blue staining

After washing, the hard tissue sections were stained with the toluidine blue solution. After uniform dyeing, dyeing was terminated, and neutral gum was applied to seal the film. The results were obtained under an inverted microscope. Bone-to-implant contact (BIC%) was counted based on the analysis results of Image-Pro Plus.

### Immunohistochemistry (IHC) assay

The samples were fixed with 4% paraformaldehyde for 48 h and dehydrated by applying 70%, 80%, 90%, 95%, and 100% ethanol. The samples were embedded and sliced into 4-μm-thick slices. The slices were dewaxed in xylene and treated with 100%-95%-90%-80% gradient alcohol. After treatment with 3% H_2_O_2_ for 15 min, the antigen was repaired by microwave thermal repair method. After sealing using normal goat serum at 37 °C for 30 min, the slices were dripped with anti-NF200 (1,100 dilution) overnight at 4 °C. After treatment with biotin-labeled goat anti-mouse IgG at 37 °C for 60 min, the sections were drip-fed with S-A/HRP for 37 °C for 30 min. After DAB staining, the slices were stained using hematoxylin for 3 min, colored with 1% hydrochloric acid alcohol for 5 s, and treated with 4% ammonia water for 5 min. After dehydration, the slices were transparent using xylene, and the immunohistochemical staining sections were observed under the microscope. The images were collected with NIS-Elements software, and the average optical density value (AOD) was analyzed with Image-Pro Plus 6.0.

### Statistical analysis

The experiment was independently repeated for 3 times, and SPSS 16.0 software was applied for statistical analysis of all experimental data. The experimental data were expressed as mean ± standard deviation (SD), and the data were corrected by two-factor analysis of variance and Bonferroni method. *P* = 0.05 was taken as the criteria for significance test.

## Results

The surface topography, surface adhesion, and ingredients were identified in HA and NGF-CS/HA coatings.

**Fig. 1:**
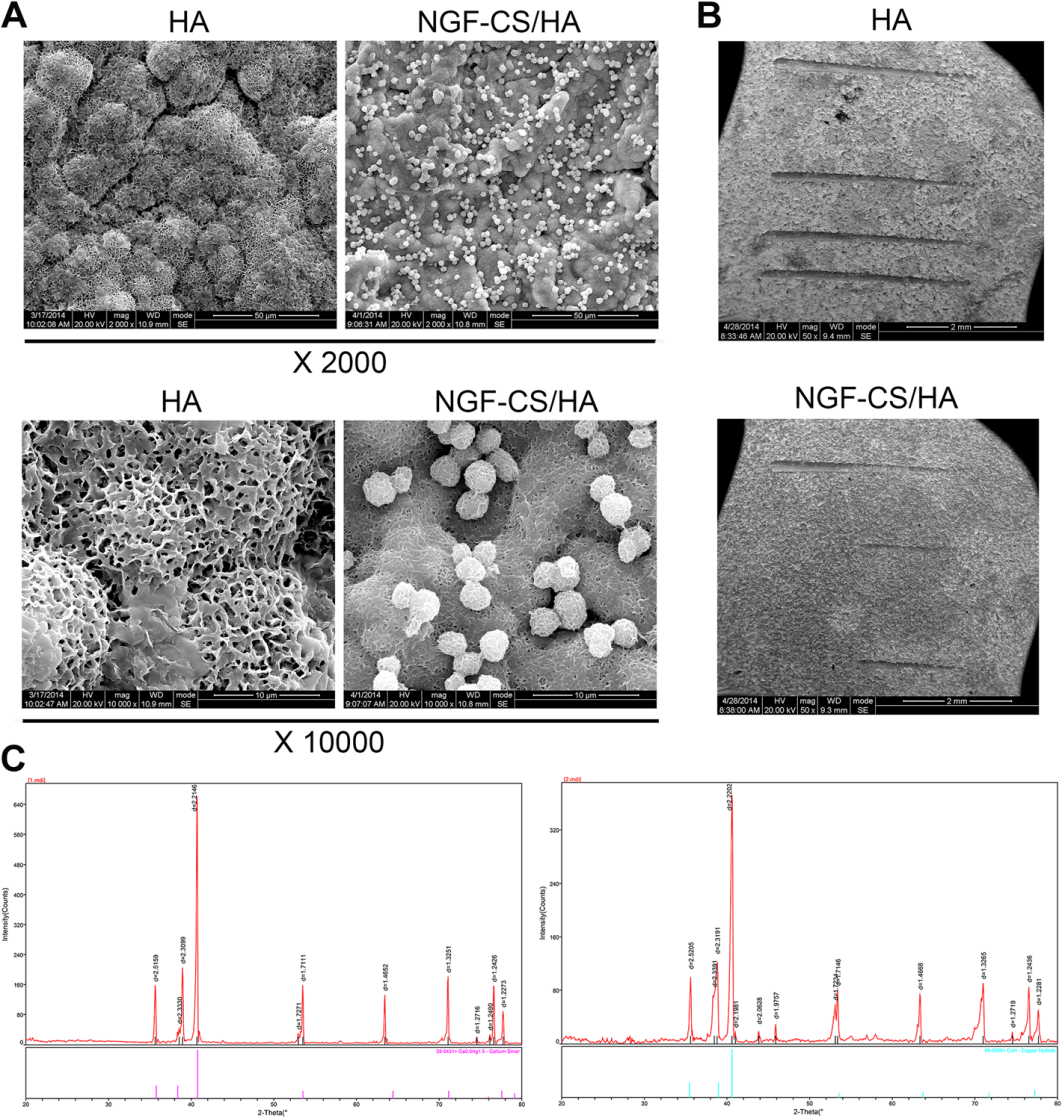
The surface topography, surface adhesion, and ingredients were identified in HA and NGF-CS/HA coatings. **a** The surface topography was observed by applying SEM in HA (*n* = 3) and NGF-CS/HA (*n* = 3) coatings. Magnification, × 2000; magnification, × 10000. **b** The scratch in NGF-CS/HA composite coatings were observed by SEM. **c** The ingredients were analyzed through XRD spectrums using the Origin7.0 software in HA (*n* = 3) and NGF-CS/HA (*n* = 3) coatings

HA and NGF-CS/HA coatings were prepared by the modified biomimetic method, and the characteristics and ingredients of HA and NGF-CS/HA coatings were identified through SEM and X-ray diffractometer. We discovered that the surfaces of HA and NGF-CS/HA coatings were porous mesh structures, and the porosity on the surface of NGF-CS/HA coating is smaller than that of the surface of HA coating; besides, the NGF-CS complex can also be uniformly deposited on or inside the HA coating (Fig. 1a). Meanwhile, we uncovered that compared to HA coating, there was no difference in the adhesion between the NGF-CS/HA coating and the sample surface (Fig. 1b). In addition, we also indicated that the main ingredients were Ti and HA on the surfaces of both HA coating and NGF-CS/HA coating through the comparison of XRD patterns (Fig. 1c).

### Implantation and removal of NGF-CS/HA-coating implants in the mandible of Beagle dogs

**Fig. 2:**
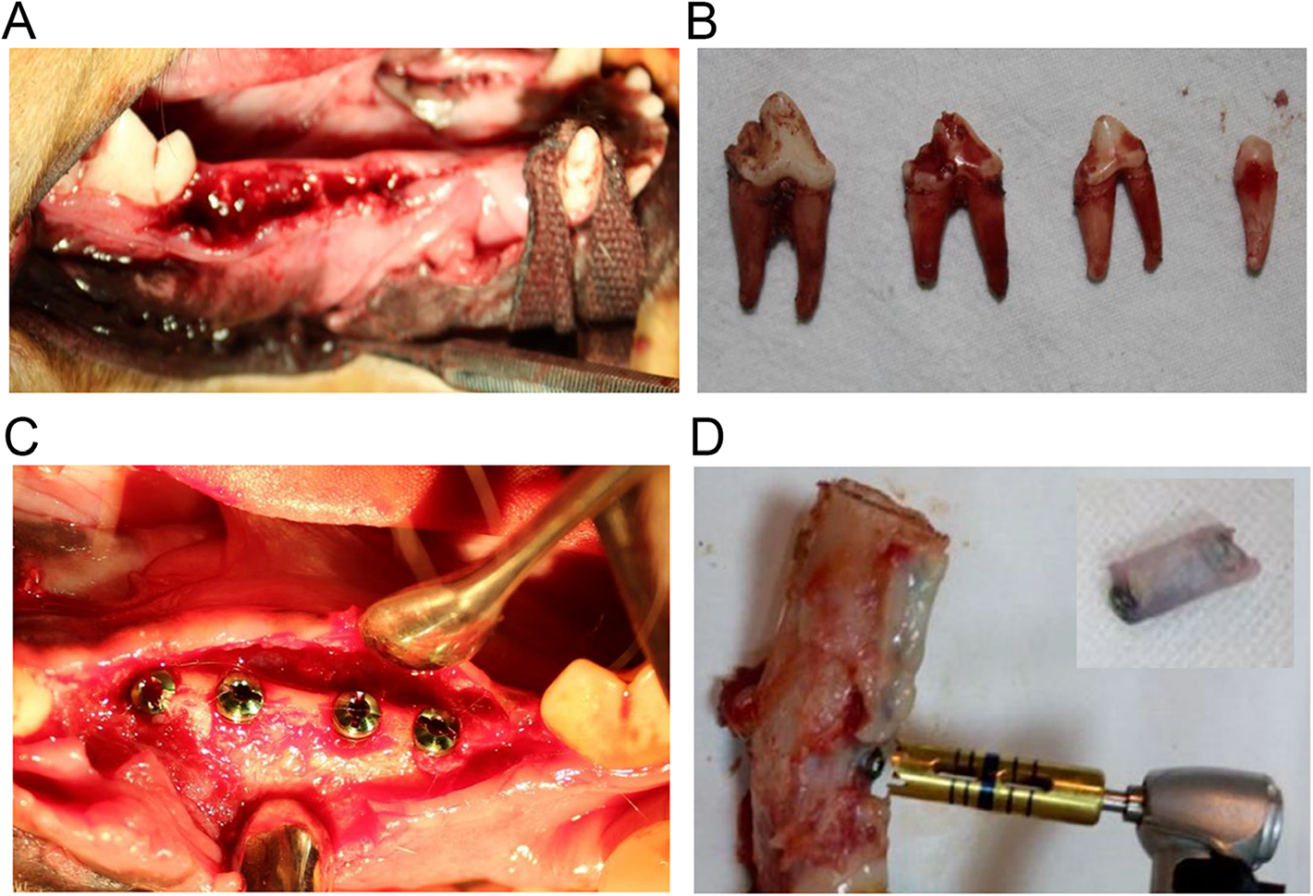
Implantation and removal of NGF-CS/HA-coating implants in the mandible of Beagle dogs. **a** The alveolar ridge of a Beagle dog after extraction. **b** The premolar teeth of a Beagle dog after extraction. **c** Six months after tooth extraction, 4 NGF-CS/HA-coating implants were implanted in the right mandibular premolar. **d** A ring bone drill was applied to remove the implants with thin layers of bone tissue

Next, we minimally extracted four bilateral mandibular premolars and tightly sutured the gums. We also exhibited the alveolar ridge and premolar teeth of Beagle dog (Fig. 2a, b). And the right mandibular premolar was implanted with HA- and NGF-CS/HA-coating implants at 6 months after tooth extraction; we also presented the oral image of the NGF-CS/HA-coating implants after implantation in a Beagle dog (Fig. 2c). When Beagle dogs were sacrificed at the observation time point, the implants with thin bone tissues in the bilateral premolar area were removed with a circular bone drill with a diameter of 4.0 (Fig. 2d).

### Identification of the interface between NGF-CS/HA-coating implants and surrounding bone tissues in the mandible of Beagle dogs

**Fig. 3:**
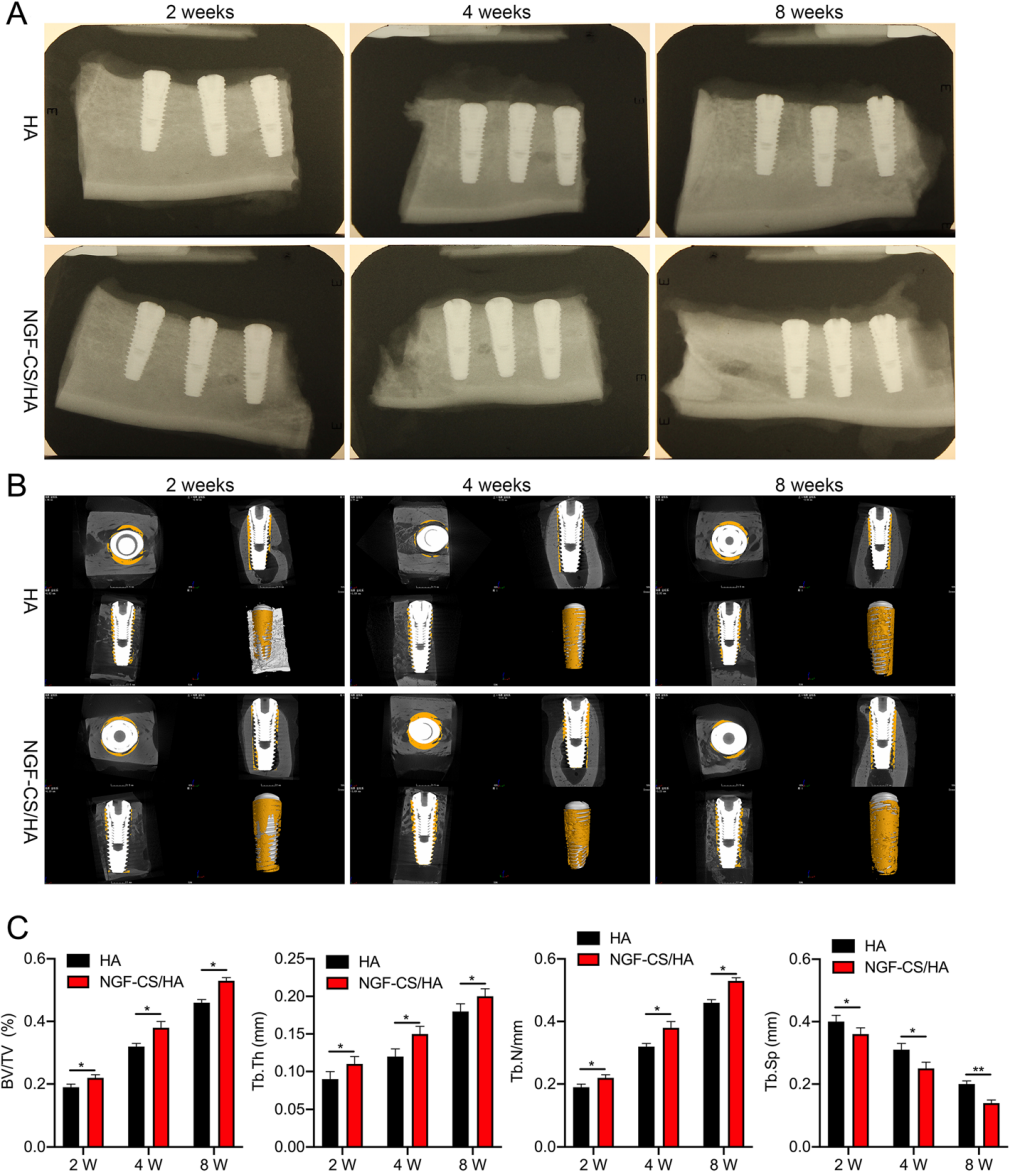
Identification of the interface between NGF-CS/HA-coating implants and surrounding bone tissues in the mandible of Beagle dogs. The interface between NGF-CS/HA-coating implants and surrounding bone tissues were observed using X-ray (**a**) and Micro-CT (**b**) at 2, 4, and 8 weeks, respectively. Two dogs with 16 implant teeth (8 teeth for HA coating and 8 teeth for NGF-CS/HA coating) at each time point

Subsequently, we further detected the characteristics of contact surfaces between NGF-CS/HA-coating implants and bone tissues through X-ray and Micro-CT at 2, 4, and 8 weeks. Firstly, we revealed that there was no obvious transmission shadow at the interface between the HA- or NGF-CS/HA-coating implant and surrounding bone tissue in the mandible of Beagle dogs at 2, 4, and 8 weeks (Fig. 3a). Secondly, we uncovered that the surfaces of both HA- and NGF-CS/HA-coating implants had bone tissue covered and uncovered areas; simultaneously, the uncovered areas in the NGF-CS/HA-coating implants were lower than those in the HA-coating implants at 2, 4, and 8 weeks, respectively; simultaneously, we revealed that compared with the 2 weeks, the uncovered areas were dramatically decreased at 4 weeks and 8 weeks, especially 8 weeks in both HA- and NGF-CS/HA-coating implant groups (Fig. 3b and Figure S1). Moreover, we proved that relative to the HA-coating implant group, BV/TV, Tb. Th, and Tb. N were prominently increased, and Tb. Sp was significantly decreased in the NGF-CS/HA-coating implant group at 2, 4, and 8 weeks, respectively. Meanwhile, BV/TV, Tb. Th, and Tb. N were gradually elevated, and Tb. Sp was gradually reduced in both HA- and NGF-CS/HA-coating implant groups with the increase of time (Fig. 3c, *P < 0.05, **P < 0.01).

### The maximum output, MAR, BIC, and nerve fiber were dramatically increased in the mandible of Beagle dogs after the implantation of NGF-CS/HA-coating implants

**Fig. 4:**
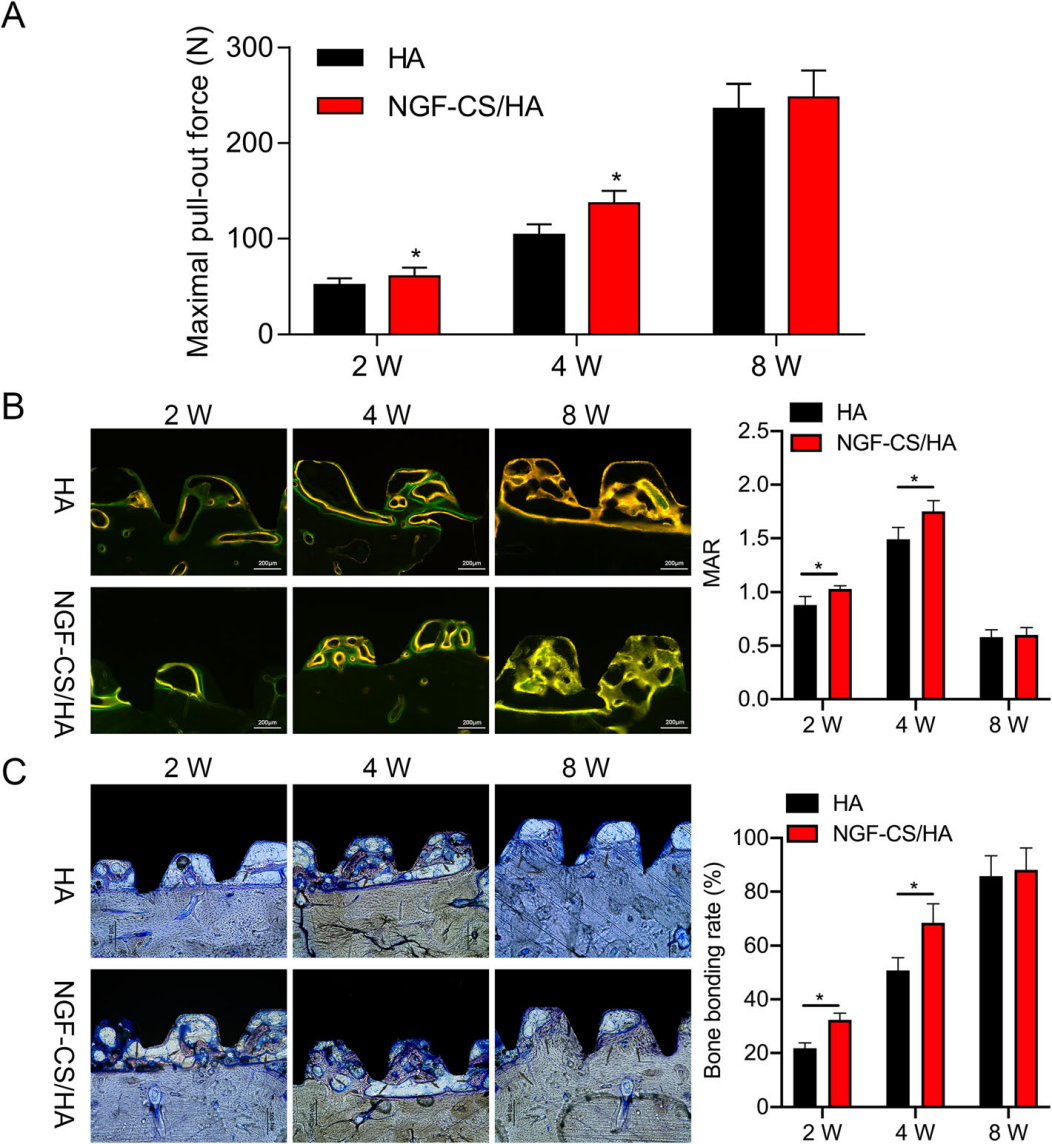
The maximum output, MAR, BIC, and nerve fiber were dramatically increased in the mandible of Beagle dogs after the implantation of NGF-CS/HA-coating implants. **a** The maximal pull-out force was applied to indicate the degree of bone bonding between NGF-CS/HA-coating implants and bone tissues at 2, 4, and 8 weeks. **b** The MAR was determined by a fluorescent microscope after labeling with alizarin red (red) and calcitrin (green) at 2, 4, and 8 weeks. Magnification, × 100; scale bar = 200 μm. **c** The new bone formation was assessed through toluidine blue staining in HA- and NGF-CS/HA-coating implant groups at 2, 4, and 8 weeks. Magnification, × 100; scale bar = 200 μm. Two dogs with 16 implant teeth (8 teeth for HA coating and 8 teeth for NGF-CS/HA coating) at each time point

Furthermore, we also explored the impacts of NGF-CS/HA-coating implants on the implant-bone bonding and peri-nerve distribution. Firstly, we applied the maximal pull-out force to represent the degree of bone bonding between NGF-CS/HA-coating implants and bone tissues at 2, 4, and 8 weeks. As exhibited in Fig. 4a, the maximal pull-out force in both HA- and NGF-CS/HA-coating implant groups was gradually enhanced with the increase of time, and the maximal pull-out force in the NGF-CS/HA-coating implant group was higher than that in the HA-coating implant group at 2 and 4 weeks (*P* < 0.05). Secondly, the fluorescence experimental results showed that the formation of new bone was bidirectional from both the bone wound surface and the implant surface. Also, through quantitative analysis, we revealed that the peri-implant MAR in both HA- and NGF-CS/HA-coating implant groups was the highest at 4 weeks and the lowest at 8 weeks, and the peri-implant MAR was significantly higher in the NGF-CS/HA-coating implant group than that in the HA-coating implant group at 2 and 4 weeks, respectively (Fig. 4b, *P* < 0.05). In addition, the toluidine blue staining results also displayed that most of the new bone grew from the original bone wound to the bottom of the thread, and the boundary between the new bone and the old bone was almost gone at 8 weeks, and there were more new bones in the NGF-CS/HA-coating implant group than in the HA group at 2 and 4 weeks; meanwhile, the quantitative analysis of peri-implant bone tissue revealed that the BIC in the NGF-CS/HA group was 32.4% and 68.5% at 2 and 4 weeks, and 1.49 and 1.34 times higher than that in the HA group, respectively (*P* < 0.05, Fig. 4c).

### NGF-CS/HA-coating implants induced the growth of nerve fibers in the peri-implant tissues

**Fig. 5:**
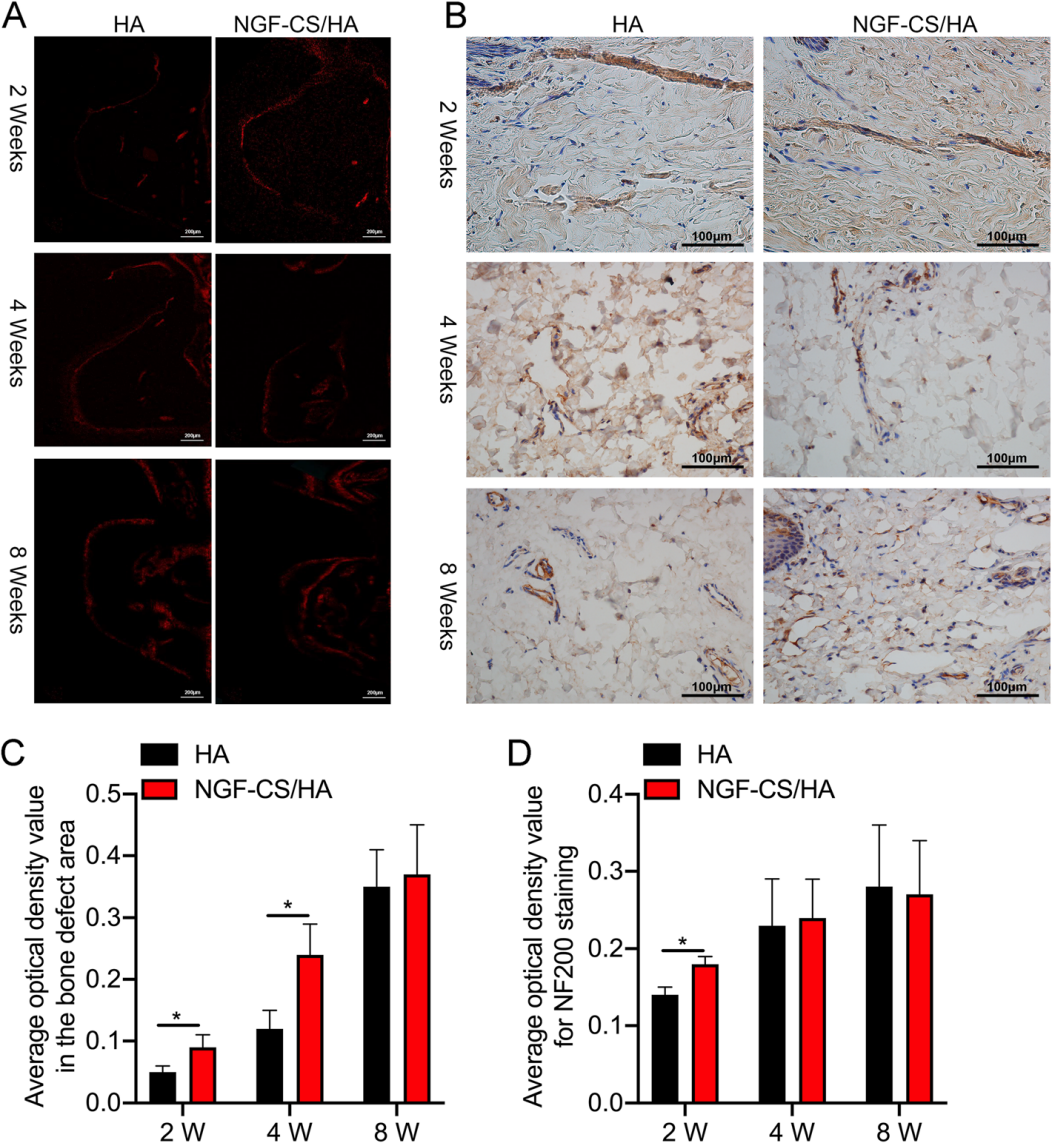
NGF-CS/HA-coating implants induced the growth of nerve fibers in the peri-implant tissues. **a** The number of nerve fiber was confirmed via DiI neural tracer in HA- and NGF-CS/HA-coating implant groups at 2, 4, and 8 weeks. Magnification, × 100; scale bar = 200 μm. **b** The expression of NF200 was monitored using IHC assay in HA- and NGF-CS/HA-coating implant groups at 2, 4, and 8 weeks. Magnification, × 400; scale bar = 100 μm. The arrows denote the longitudinal section of the nerve fibers. Two dogs with 16 implant teeth (8 teeth for HA coating and 8 teeth for NGF-CS/HA coating) at each time point

Besides, DiI neural tracer analysis also testified that the peri-implant regeneration of nerves was from the inferior alveolar nerve of the mandibular nerve, which was mainly located in the trapezoidal bone defect area and the bone marrow (Fig. 5a). Meanwhile, the average optical density (AOD) value of red development in the defect area of implant pitch trapezoidal bone was analyzed by Image-Pro Plus 6.0, and we found that the AOD was gradually raised in both HA- and NGF-CS/HA-coating implant groups with the increase of time, and AOD was also memorably elevated in the NGF-CS/HA-coating implant group compared with that in the HA group at 2 weeks and 4 weeks, respectively (*P* < 0.05, Fig. 5c). Our data also presented that the clay bank or brownness indicated the NF200-positive cells, and the irregularly distributed nerve fibers and bundles could be observed in the gingiva of the two implant groups. Through quantitative analysis, we proved that the AOD of NF200 staining was gradually increased in both HA- and NGF-CS/HA-coating implant groups with the increase of time. Also, the AOD of NF200 staining was significantly increased in the NGF-CS/HA-coating implant group with respect to that in the HA group (*P* < 0.05, Fig. 5b, d).

### The implantation of NGF-CS/HA-coating implants markedly upregulated the levels of NGF-, osteogenesis differentiation-, and neurogenic differentiation-related genes

**Fig. 6:**
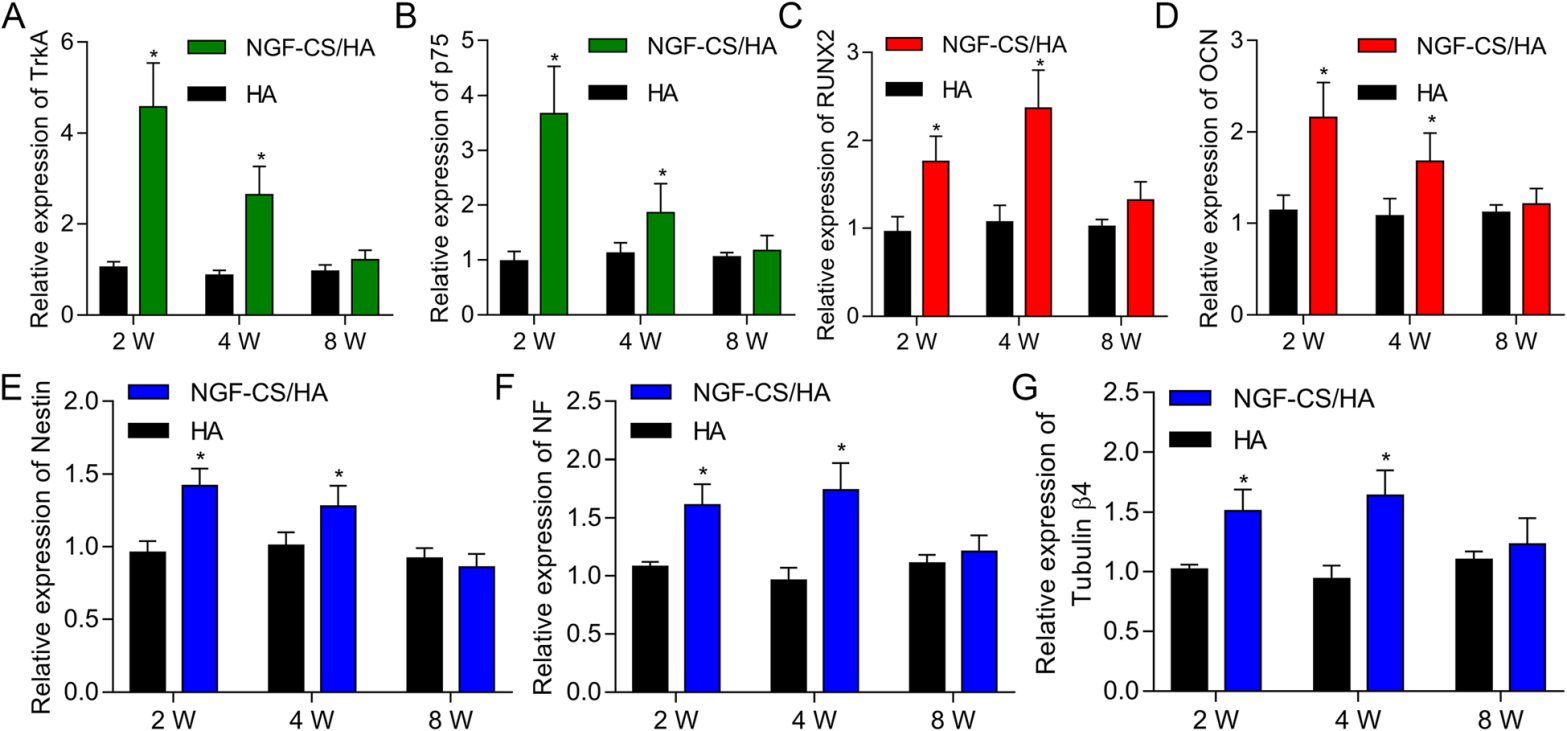
The implantation of NGF-CS/HA-coating implants markedly upregulated the levels of NGF-, osteogenesis differentiation-, and neurogenic differentiation-related genes. RT-qPCR analysis of Trk A and p75 (**a**, **b**); OCN and Runx-2 (**c**, **d**); and Nestin, NF, and tubulin β4 (**e**–**g**) in HA- and NGF-CS/HA-coating implant groups at 2, 4, and 8 weeks. *P < 0.05. Two dogs with 16 implant teeth (8 teeth for HA coating and 8 teeth for NGF-CS/HA coating) at each time point

To further investigate the influences of NGF-CS/HA-coating implants on the osteogenic and neurogenic differentiation in the mandible of Beagle dogs, IHC and RT-qPCR assays were conducted. Moreover, we disclosed that the levels of NGF-related genes (Trk A and p75), osteogenesis differentiation-related genes (OCN and Runx-2), and neurogenic differentiation-related genes (Nestin, tublin β-4, and NF) were remarkably reduced in the NGF-CS/HA-coating implant group at 2 and 4 weeks, respectively. Also, Runx-2 and tubulin β-4 expressions were significantly higher in the NGF-CS/HA-coating implant group than those in the HA-coating implant group at 2 and 4 weeks, respectively (*P* < 0.05, Fig. 6).

## Discussion

The biochemical modification of the implant surface is to promote the occurrence and development of bone bonding by immobilizing specific biomolecules on the implant surface [46–48]. NGF, as a polyprotein, is widely distributed in various tissues and organs [49]. Research has proved that NGF has the capacity to facilitate fracture healing [50]. NGF can also accelerate endothelial cell proliferation, related gene expression, and angiogenesis on different titanium surfaces [51–53]. Besides, NGF has anti-inflammatory effects and can induce early osseointegration in peri-implant tissues [54, 55]. In our study, we prepared the NGF-CS/HA-coating implants by depositing NGF-CS complex and HA on the surface of titanium in the high concentration of SBF. And we revealed that the surface of NGF-CS/HA-coating implants presented a porous mesh structure, and the Ti and HA were the main ingredients on the surfaces of both HA and NGF-CS/HA coatings.

The width and sclerotin of a Beagle dog are close to that of human alveolar bone, which has become an ideal animal for the study of implant surface modification [56]. The orthotopic model was established in the alveolar ridge of Beagle dogs, which is more similar to the human oral condition [57]. The Beagle dog’s mandibular premolars were pulled out, and the HA- and NGF-CS/HA-coating implants were successfully implanted on both sides of the mandible in Beagle dogs after the tooth extraction wound healed completely 6 months later. Some researches proved that the bone microstructure is closely related to its biomechanical properties and has a certain influence on bone strength [58, 59]. In order to prevent coating stripping, the implant was implanted under the condition of torque ≤ 20 N cm in combination with the results of adhesion force, and we discovered that no implant loosening occurred after the operation. And the toluidine blue staining results exhibited no obvious HA-coating peeling phenomenon, indicating that the animal model of Beagle dog mandible implanted with NGF-CS/HA coating was successfully established. Besides, we demonstrated that NGF-CS/HA-coating implants prominently increased the BV/TV, Tb. Th, and Tb. N and significantly decreased Tb. Sp, suggesting that NGF-CS/HA-coating implants could improve the trabecular microstructure.

Moreover, we also revealed that the capacities of osseointegration and bone regeneration were dramatically enhanced in the peri-implant tissues of Beagle dogs after the implantation of NGF-CS/HA-coating implants. More and more researches certified that functional neuroreceptors were existed in the bone tissue around the implants, which plays a role of replacing the proprioceptor of the parodontium [60, 61]. In our study, we also disclosed that the peri-implant nerves were mainly derived from the inferior alveolar nerves and located in the trapezoidal bone defect area and the bone marrow. And the irregularly distributed nerve fibers and bundles could be significantly increased in the gingival of Beagle dogs after the implantation of NGF-CS/HA-coating implants. In the process of nerve growth and repair, NGF can improve the survival rate of nerve cells, promote the growth of nerve protrusion, induce the directional growth of protuberances, and determine the direction of nerve fibers. Therefore, we speculated that the NGF-CS/HA composite coatings could promote the osseointegration of the implants and increase the number of peri-implant nerves, and the NGF also indirectly promotes the osseointegration and the regulation of nerve to bone tissues; with the increase of healing time, the release of NGF was significantly reduced, and the indirect role of promoting osseointegration was also gradually weakened. In accordance with reports in the literatures, we discovered that the modification of multiple Ti dental implants contributes to the peri-implant tissues. For example, different shapes of nanostructured ceria-coated Ti surfaces could improve the antibacterial and anti-inflammatory properties of dental implants [62]; tantalum-modified Ti implants have also proven to have good superior bacteriostasis and osseointegration [63]; silanization-modified Ti implants could dramatically improve bone resorption induced by peri-implantitis in Beagle dogs [64]; minocycline hydrochloride-loaded graphene oxide (GO)-modified Ti implants also have certain therapeutic action on the peri-implantitis in Beagle dogs [65]. However, these modified Ti dental implants are only proven to be antibacterial, inhibit inflammation, or improve bone absorption in peri-implant tissues. Our current study demonstrated that NGF-CS/HA coating not only induced osseointegration of implants, but also enhances peripheral nerve regeneration.

However, there are still some limitations in the current study, which will be further explored in our future studies. For instance, the number of animals is small, which should be added in a subsequent study; the physiological relevance of the time points should also be further explored; the adhesion between implant and coating has not reached the level of direct clinical application; the mechanism of NGF-CS/HA composite coating in the osseointegration and peri-implant nerve regeneration also needs to be further investigated; it is also required to explore the regulation mechanism of NGF-CS/HA-coating implants on the relevant genes (Trk A, p75, OCN, Runx-2, Nestin, tublin β-4, NF, Runx-2, and tubulin β-4) and cellular molecular pathway.

## Conclusion

We proved that NGF-CS/HA coating could significantly accelerate the implant osseointegration and enhance the regeneration of peri-implant nerves, which might provide a certain experimental basis for the application of NGF-CS/HA-coating implants in oral implants.

## Supplementary Information

**Additional file 1: Figure S1** The differences in the amount of ‘uncovered’ area between the HA and NGF-CS/HA groups. * *P* < 0.01. Two dogs with 16 implant teeth (8 teeth for HA coating and 8 teeth for NGF-CS/HA coating) at each time point.

## Data Availability

The datasets used and/or analyzed during the current study are available from the corresponding author on reasonable request.

## Abbreviations

NGF: Nerve growth factor
CS: Chondroitin sulfate
HA: Hydroxyapatite
SBF: Simulated body fluids
SLA: Sandblast-acid erosion
ROI: Range of interest
BVF: Bone volume fraction
Tb.Th: Trabecular thickness
Tb.N: Trabecular number
Tb.Sp: Trabecular spacing
