# Supplementary figures and images for: Nerve growth factor-chondroitin sulfate/hydroxyapatite-coating composite implant induces early osseointegration and nerve regeneration of peri-implant tissues in Beagle dogs
Source: J Orthop Surg Res. 2021 Jan 13;16:51. doi: 10.1186/s13018-020-02177-5 (PMC7805124; doi:10.1186/s13018-020-02177-5)

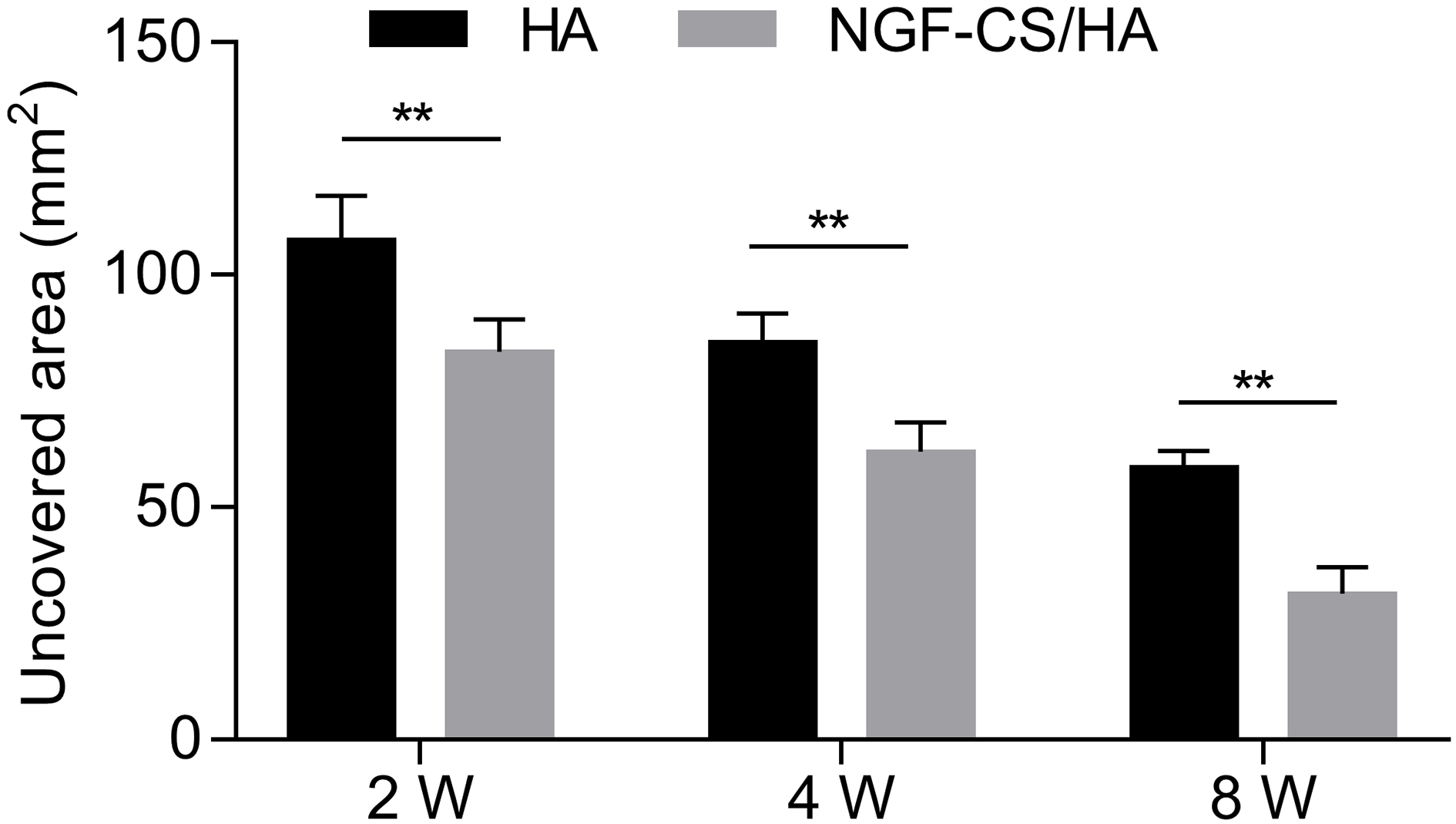

Supplement: Supplementary file 1 — Additional file 1: Figure S1 The differences in the amount of ‘uncovered’ area between the HA and NGF-CS/HA groups. * P < 0.01. Two dogs with 16 implant teeth (8 teeth for HA coating and 8 teeth for NGF-CS/HA coating) at each time point. [file 13018_2020_2177_MOESM1_ESM.tif]
